# Crystal structure of bis(3,5-dichloro-2-hydroxybenzyl)(2-methoxyethyl)amine

**DOI:** 10.1107/S2056989023006564

**Published:** 2023-08-04

**Authors:** Bradley M. Wile, Claire L. Griffith, Adam R. Johnson

**Affiliations:** a The Donald J. Bettinger Department of Chemistry and Biochemistry, The School of Science, Technology, and Mathematics, Ohio Northern University, 525 S. Main Street, Ada, OH 45810, USA; b Harvey Mudd College, Chemistry, 301 Platt Blvd., Claremont, CA 91711, USA; Purdue University, USA

**Keywords:** crystal structure, phenol, amine, ether, amine­bis­(phenol)

## Abstract

The title compound, systematic name 4,4′,6,6′-tetrachloro-2,2′-{[(2-methoxy­ethyl)azanediyl]bis(methylene)}diphenol (C_17_H_17_Cl_4_NO, **1**), was prepared *via* a modified Mannich reaction between 2-meth­oxy­ethyl­amine, 2,4-di­chloro­phenol, and aqueous formaldehyde. The resulting amine bis­(phenol) provides an inter­esting comparison to related species as a result of the electron-withdrawing substituents on the phenol rings, in combination with similar steric parameters. One of the Cl atoms was modeled as a two-component disorder with partial occupancies of 0.49 (3) and 0.51 (3), while the pendant ether group was modeled as a two-component disorder with partial occupancies of 0.867 (3) and 0.133 (3). A comparison of metrical parameters for the title compound and closely related structures provides insight into the use of these species as ligands to support transition-metal complexes for applications as homogeneous catalysts.

## Abstract 2

The title compound was prepared *via* a modified Mannich reaction between 2-meth­oxy­ethyl­amine, 2,4-di­chloro­phenol, and aqueous formaldehyde. The resulting amine bis­(phenol) provides an inter­esting comparison to related species as a result of the electron-withdrawing substituents on the phenol rings, in combination with similar steric parameters.

## 1. Chemical context

Complexes of early transition- and rare-earth metals featuring di­amine­bis­(phenols) have been employed as efficient catalysts for the polymerization of olefins and cyclic esters (Tshuva *et al.*, 2000 ▸; Carpentier *et al.*, 2015 ▸), while those of late transition metals have been shown to be effective at promoting cross-coupling (Hasan *et al.*, 2011 ▸; Qian *et al.*, 2011 ▸; Reckling *et al.*, 2011 ▸). Several reports have noted that the coordination mode and donor-atom identity play an important role in the activity of the resulting complexes (Tshuva *et al.*, 2001 ▸; Qian *et al.*, 2011 ▸; Chard *et al.*, 2014 ▸). We have previously observed both κ^2^ and κ^3^ coordination modes for Pd^II^ complexes of related amine­bis­(phenols), in which steric parameters of the phenolate moiety played a significant role in the coordination behavior (Graziano, Collins *et al.*, 2019 ▸; Graziano, Wile *et al.*, 2019 ▸).

Di­amine­bis­(phonols) may be readily prepared *via* a Mannich reaction (Tshuva *et al.*, 2000 ▸, 2001 ▸; Kasting *et al.*, 2015 ▸), and the ligand framework may be modified by altering the steric or electronic parameters of the commercially available reaction components. Both bridging and pendant di­amine variants are known, depending on whether the ligand precursor is prepared using an N,N- or N,N′-disubstituted amine. Prior reports of Fe^II^ complexes supported by halogenated amine­bis­(phenols) bearing an alkyl ether donor group suggest poorer catalytic activity when compared with ligands bearing bulky alkyl-substituted phenols (Hasan *et al.*, 2011 ▸; Reckling *et al.*, 2011 ▸). However, it is speculated that the inferior catalytic activity is related to the air sensitivity of these Fe complexes, and potential catalyst decomposition pathways under the conditions employed for this Kumada coupling. Based on these reports and our inter­est in extending the range of amine­bis­(phenols) suitable for use as ligands, we prepared the title compound **1** and obtained single crystals suitable for X-ray diffraction studies. It was speculated that a direct comparison of the metrical parameters for **1** with those of related amine­bis­(phenols) with pendant ether groups would provide insight into the choice of halogenated phenols in the design of this ligand, for use in combination with late transition metals. 
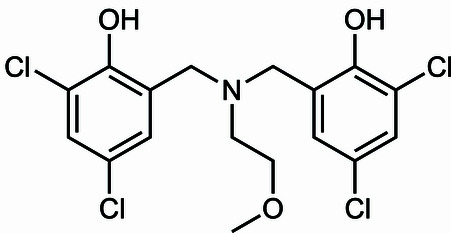


## 2. Structural commentary

**Figure 1:**
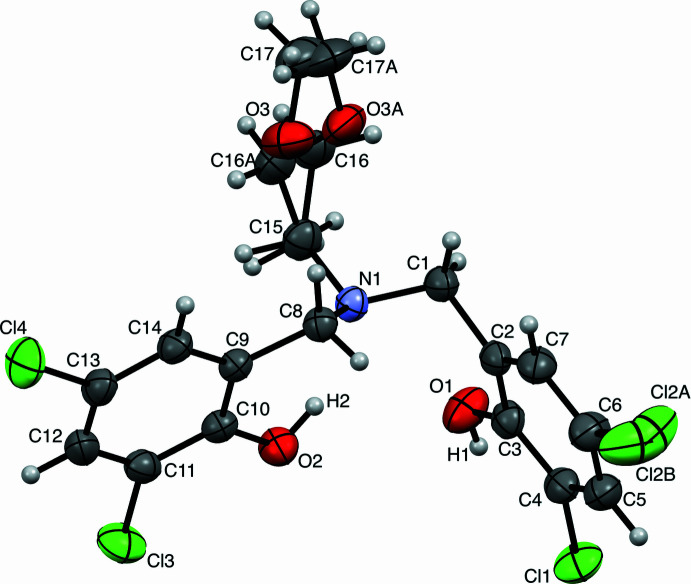
Labeled depiction of the title compound, with displacement ellipsoids drawn at the 50% probability level.

Compound **1** exhibits bond lengths and angles consistent with the depiction as an aliphatic amine with ether and phenol moieties depicted in Fig. 1 ▸. C—N bond lengths [1.472 (2), 1.475 (2), and 1.476 (2) Å], C—O_phenol_ bond lengths [1.354 (1) and 1.346 (2) Å], and C—Cl bond lengths [1.734 (1), 1.732 (1), 1.728 (1), and 1.732 (9) Å] are not significantly different from one another, within ±3 esd. The sum of C—N—C angles about N1 is 337.7°, representing a deviation of 0.41093 (1) Å from the plane defined by C1/C8/C15. This extent of pyramidalization is consistent with an *sp* ^3^-hybridized (tertiary) amine, and is influenced by the presence of an intra­molecular O2—H2⋯N1 hydrogen bond (Table 1 ▸). This hydrogen-bonding inter­action generates a six-membered ring with an *S*(6) graph-set descriptor, consistent with our prior observations for similar mol­ecules (Bowser *et al.*, 2016 ▸). The ether fragment was modeled as a two-component disorder involving C16, O3, and C17, with a C16—O3—C17 bond angle of 112.0 (3)°. The methyl­ene [C16—O3, 1.405 (3) Å] and methyl [C17—O3, 1.413 (4) Å] C—O bond lengths are not significantly different from one another, within ±3 esd. Atom Cl2 was also modeled as having two-component disorder.

Compound **1** is chemically similar to the related ligands featuring alkyl substituents in place of the Cl substituents in **1**. A comparison of bond lengths and angles for compound **1** and CAKDUP (Hasan *et al.*, 2011 ▸), ZAVTEX (Dean *et al.*, 2012 ▸), SOJBIE and SOJBUQ (Chapurina *et al.*, 2014 ▸) is presented in Table 2 ▸. Despite the differences in space group, all compounds exhibit similar metrical parameters. The most notable differences between these structures are the shorter C—O_phenol_ bond lengths for compound **1** [1.354 (1) and 1.346 (2) Å], consistent with the electron-withdrawing effect of the Cl substituents on the phenol rings. In contrast, compounds containing electron-donating alkyl substituents exhibit slightly longer C—O_phenol_ bond lengths. Bond lengths for other moieties are more similar between **1** and these previously reported structures. The sum of C—N—C bond angles and the C—O—C bond angles indicate a similar electronic environment for the amine and ether donors of all compounds. This supports the hypothesis that compound **1** would have similar steric parameters to closely related ligands, but function as a more electrophilic donor.

## 3. Supra­molecular features

**Figure 2:**
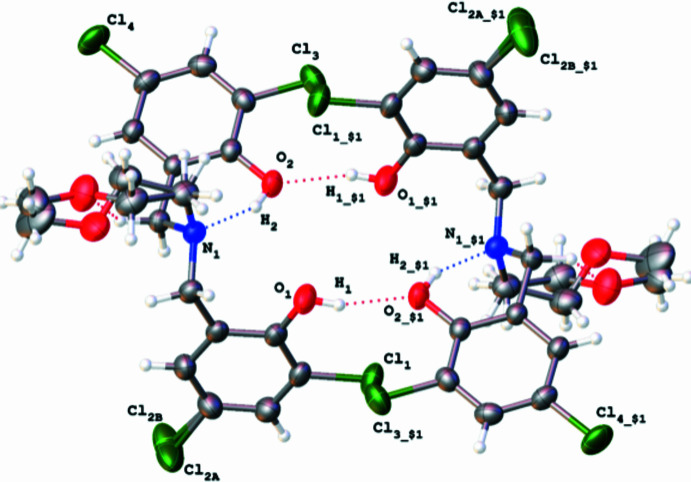
Depiction of the centrosymmetric dimer formed as a result of hydrogen bonding. See Table 1 ▸ for symmetry codes.

The hydrogen-bond geometry is noted in Table 1 ▸. A short contact was noted between O2 and N1 [2.6365 (14) Å], consistent with a hydrogen bond between the phenol and tertiary amine moieties, O2—H2⋯N1. By refining the position of H2, the H2⋯N1 distance was found to be 1.83 (2) Å, suggesting a strong hydrogen-bonding inter­action that supports the observed pyramidalization of the tertiary amine. Hydrogen bonding is also observed between O1—H1⋯O2′ (and conversely O1′—H1′⋯O2), resulting in the formation of a centrosymmetric dimer with an 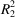 (20) graph-set descriptor, as shown in Fig. 2 ▸. The H1⋯O2′ distance [2.01 (2) Å] suggests a strong hydrogen-bonding inter­action.

An additional short contact was noted between O1 and Cl1 [3.0459 (12) Å] with a corresponding H1⋯Cl1 distance of 2.58 (2) Å, suggesting a weak inter­action. Close contacts between Cl1⋯Cl4′ [3.468 (3) Å] and Cl1⋯O2′ [3.266 (2) Å] centers are inconsistent with weak halogen bonding, and instead are attributed to packing effects. Further evidence is provided by the small observed angles around Cl1 (104.10° for C4—Cl1⋯O2′ and 72.24° for C4—Cl1⋯Cl4′) and Cl4 (147.46° for C13—Cl4⋯Cl1′) compared with 180° expected for a halogen bond.

## 4. Database survey

A search of the Cambridge Structural Database (CSD, update of November 2022; Groom *et al.*, 2016 ▸) for related amine bis­(phenols) featuring a pendant ether moiety returned 19 results, all featuring alkyl or hydrogen substituents on the phenol. Of these, the most closely related were reported by Kozak and co-workers, and feature 2,4-dimethyl or 2-*tert*-butyl-4-methyl phenol substituents in place of the Cl substit­uents reported in this work. These include CSD refcodes CAKDUP (Hasan *et al.*, 2011 ▸), HITHIC (Chowdhury *et al.*, 2008 ▸), and ZAVTEX (Dean *et al.*, 2012 ▸). Structures XAQWUL, XAQXAS, XARCOM, XARCUS, XARDAZ, and XARHOR (Fazekas *et al.*, 2021 ▸) are derived from various amino-acid ethyl esters and feature 2,4-dimethyl or 2,4-di-*tert*-butyl substituents. TIDLIC (Safaei *et al.*, 2007 ▸) features a similar 2,4-di-*tert*-butyl substitution pattern in combination with a pendant methyl-tetra­hydro­furanyl amine substituent. Structure UZOZOA (Kuźnik *et al.*, 2019 ▸) contains a di­eth­oxy­ethyl amine moiety as well as otherwise unsubstituted 2-naphthol donors as a synthetic precursor to the target ligand. Structures SOJBIE and SOJBUQ (Chapurina *et al.*, 2014 ▸) featuring bulky cumyl substituents were reported as synthetic precursors to the corresponding Sc and Y complexes.

A series of compounds featuring amino phenols as part of a larger structure or macrocycle have been reported. KEWFUP, KEWGAW, and KEWGEA (Riisiö *et al.*, 2012 ▸) feature two amine-bis­(phenol) moieties connected by an ethyl-bis­(eth­oxy­eth­yl) linkage and exhibit significant hydrogen bonding in the solid state. Two related macrocycles featuring an ethyl-bis­(eth­oxy­eth­yl) PEXNOY (Takemura *et al.*, 2018 ▸) or di­sulfide MEQFUJ (Ito *et al.*, 2000 ▸) bridge have been reported. Entry TAXLIN (Hampton *et al.*, 1996 ▸) is a tri-aza-calix[3]arene featuring a glycine-derived amino ester moiety.

## 5. Synthesis and crystallization

**Figure 3:**
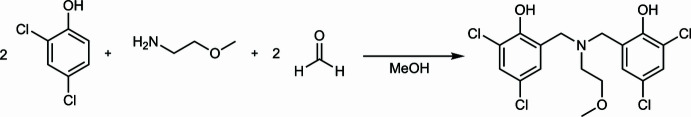
Reaction scheme.

Compound **1** was prepared using a method analogous to that reported for related compounds (Graziano, Collins *et al.*, 2019 ▸; Reckling *et al.*, 2011 ▸). This reaction scheme is shown in Fig. 3 ▸. 2,4-Di­chloro­phenol (1.957 g, 12.0 mmol, 2 eq.) and a 37 wt.% aqueous solution of formaldehyde (0.974 g, 12.0 mmol, 2 eq.) were added to a 20 mL scintillation vial containing 5.0 mL of methanol and a PTFE-coated magnetic stir bar. 2-Meth­oxy­ethyl­amine (0.521 mL, 6.00 mmol, 1 eq.) was added, and the vial was immediately capped and placed in an aluminum heating block maintained at 343 K. The clear colorless solution turned bright yellow within 1 h of heating, and maintained this appearance for 18 h, at which time the vial was removed from the heating block. The reaction mixture was poured into cold water (20 mL), and extracted with ethyl acetate (3 × 20 mL). The organic layers were combined, dried over MgSO_4_, and concentrated *in vacuo* to yield a yellow oil. The product was purified using an automated column chromatography system with an ethyl acetate/hexa­nes gradient (0% EtOAc hold 1 min → 20% EtOAc in 2 min, hold 4 min → 100% EtOAc in 4 min, hold 2 min). The desired product was isolated as a yellow oil (0.594 g, 1.40 mmol, 23%, *R*_f_ = 0.40 in 40% EtOAc) that generated single crystals suitable for X-ray diffraction studies upon standing.

## 6. Refinement

Crystal data, data collection and structure refinement details are summarized in Table 3 ▸. Atoms H1 and H2 were located in difference-Fourier maps and freely refined. All other hydrogen atoms were placed at calculated positions (aromatic: 0.93 Å, methyl­ene: 0.97 Å, meth­yl: 0.96 Å) using suitable HFIX commands and refined as riding with *U*_iso_(H) = 1.2–1.5*U*_eq_(C). The methyl group was refined as an idealized rotating group. Cl2 was modeled as a two-component disorder with partial occupancies of 0.49 (3) and 0.51 (3). The pendant ether group was modeled as a two-component disorder with partial occupancies of 0.867 (3) and 0.133 (3). Atomic displacement parameters were restrained using SIMU with a sigma of 0.01 for inter­nal and 0.02 for terminal atoms. The atoms within the disordered group were restrained to have similar bond distances. Cl2 was modeled as a two-component disorder with partial occupancies of 0.52 (4) and 0.48 (4).

## Supplementary Material

Crystal structure: contains datablock(s) global. DOI: 10.1107/S2056989023006564/zl5047sup1.cif

CCDC reference: 2067395

Additional supporting information:  crystallographic information; 3D view; checkCIF report

## Figures and Tables

**Table 1:** Hydrogen-bond geometry (Å, °)

*D*—H⋯*A*	*D*—H	H⋯*A*	*D*⋯*A*	*D*—H⋯*A*
O1—H1⋯Cl1	0.82 (2)	2.58 (2)	3.0455 (12)	117.4 (17)
O2—H2⋯N1	0.87 (2)	1.83 (2)	2.6365 (16)	153 (2)
O1—H1⋯O2^i^	0.82 (2)	2.01 (2)	2.7709 (14)	152.5 (19)

Symmetry code: (i) 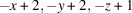 .

**Table 2:** Comparison of bond lengths (Å) and sum of angles (°) for **1** and related structures

	**1**	CAKDUP	ZAVTEX	SOJBIE	SOJBUQ
C—O(phenol)	1.352 (2)	1.370 (1)	1.369 (3)	1.369 (2)	1.368 (3)
	1.348 (3)	1.375 (2)	1.370 (3)	1.370 (2)	1.370 (3)
C—O—C	112.0 (3)	112.5 (1)	114.5 (2)	112.2 (1)	112.1 (2)
ΣC—N—C	337.7	334.0	334.2	333.5	333.4

**Table 3:** Experimental details

Crystal data	
Chemical formula	C_17_H_17_Cl_4_NO_3_
*M*_r_	425.11
Crystal system, space group	Triclinic, *P* 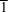
Temperature (K)	293
*a*, *b*, *c* (Å)	9.4912 (2), 10.0464 (2), 11.1169 (3)
α, β, γ (°)	103.930 (2), 113.048 (2), 90.543 (2)
*V* (Å^3^)	940.32 (4)
*Z*	2
Radiation type	Mo *K*α
μ (mm^−1^)	0.65
Crystal size (mm)	0.56 × 0.36 × 0.31

Data collection	
Diffractometer	XtaLAB Mini II
Absorption correction	Analytical (*CrysAlis PRO*; Rigaku OD, 2019 ▸)
*T*_min_, *T*_max_	0.886, 0.940
No. of measured, independent and observed [*I* > 2σ(*I*)] reflections	56643, 5724, 4418
*R*_int_	0.056
(sin θ/λ)_max_ (Å^−1^)	0.722

Refinement	
*R*[*F* ^2^ > 2σ(*F* ^2^)], *wR*(*F* ^2^), *S*	0.035, 0.100, 1.05
No. of reflections	5724
No. of parameters	274
No. of restraints	71
H-atom treatment	H atoms treated by a mixture of independent and constrained refinement
Δρ_max_, Δρ_min_ (e Å^−3^)	0.34, −0.25

Computer programs: *CrysAlis PRO* 1.171.40.55a (Rigaku OD, 2019 ▸), *SHELXT* (Sheldrick, 2015*a* ▸), *SHELXL2016/6* (Sheldrick, 2015*b* ▸), *OLEX2* 1.3-ac4 (Dolomanov *et al.*, 2009 ▸), *PLATON* (Spek, 2020 ▸).

## supplementary crystallographic information

### Crystal data

**Table:**

C_17_H_17_Cl_4_NO_3_	*Z* = 2
*M**_r_* = 425.11	*F*(000) = 436
Triclinic, *P*1	*D*_x_ = 1.501 Mg m^−^^3^
*a* = 9.4912 (2) Å	Mo *K*α radiation, λ = 0.71073 Å
*b* = 10.0464 (2) Å	Cell parameters from 18216 reflections
*c* = 11.1169 (3) Å	θ = 2.1–29.4°
α = 103.930 (2)°	µ = 0.65 mm^−^^1^
β = 113.048 (2)°	*T* = 293 K
γ = 90.543 (2)°	Block, light yellow
*V* = 940.32 (4) Å^3^	0.56 × 0.36 × 0.31 mm

### Data collection

**Table:**

XtaLAB Mini II diffractometer	4418 reflections with *I* > 2σ(*I*)
Detector resolution: 10.0000 pixels mm^-1^	*R*_int_ = 0.056
ω scans	θ_max_ = 30.9°, θ_min_ = 2.1°
Absorption correction: analytical (CrysAlisPro; Rigaku OD, 2019)	*h* = −13→13
*T*_min_ = 0.886, *T*_max_ = 0.940	*k* = −14→14
56643 measured reflections	*l* = −15→15
5724 independent reflections	

### Refinement

**Table:**

Refinement on *F*^2^	Primary atom site location: dual
Least-squares matrix: full	Hydrogen site location: mixed
*R*[*F*^2^ > 2σ(*F*^2^)] = 0.035	H atoms treated by a mixture of independent and constrained refinement
*wR*(*F*^2^) = 0.100	*w* = 1/[σ^2^(*F*_o_^2^) + (0.0501*P*)^2^ + 0.1137*P*] where *P* = (*F*_o_^2^ + 2*F*_c_^2^)/3
*S* = 1.05	(Δ/σ)_max_ = 0.001
5724 reflections	Δρ_max_ = 0.34 e Å^−^^3^
274 parameters	Δρ_min_ = −0.25 e Å^−^^3^
71 restraints	

### Special details

**Table:**

Geometry. All esds (except the esd in the dihedral angle between two l.s. planes) are estimated using the full covariance matrix. The cell esds are taken into account individually in the estimation of esds in distances, angles and torsion angles; correlations between esds in cell parameters are only used when they are defined by crystal symmetry. An approximate (isotropic) treatment of cell esds is used for estimating esds involving l.s. planes.

### Fractional atomic coordinates and isotropic or equivalent isotropic displacement parameters (Å^2^)

**Table:**

	*x*	*y*	*z*	*U*_iso_*/*U*_eq_	Occ. (<1)
Cl1	1.01005 (4)	1.16885 (4)	0.82889 (4)	0.05322 (11)	
Cl3	1.22323 (4)	0.61557 (4)	0.38567 (4)	0.05383 (11)	
Cl4	0.84093 (5)	0.14540 (4)	0.20134 (5)	0.06363 (13)	
O1	0.82934 (14)	1.03365 (11)	0.52755 (11)	0.0510 (3)	
H1	0.900 (2)	1.097 (2)	0.568 (2)	0.069 (6)*	
O2	0.96180 (11)	0.74608 (9)	0.42712 (10)	0.0400 (2)	
H2	0.872 (2)	0.774 (2)	0.414 (2)	0.074 (6)*	
N1	0.66091 (12)	0.74842 (10)	0.34291 (10)	0.0318 (2)	
C1	0.57399 (14)	0.84027 (13)	0.40526 (13)	0.0367 (3)	
H1A	0.474597	0.791182	0.382398	0.044*	
H1B	0.555600	0.919147	0.367423	0.044*	
C2	0.65628 (14)	0.89107 (12)	0.55747 (13)	0.0341 (2)	
C3	0.78319 (14)	0.99260 (12)	0.61437 (13)	0.0354 (3)	
C4	0.85263 (14)	1.04400 (13)	0.75464 (14)	0.0376 (3)	
C5	0.80028 (16)	0.99664 (15)	0.83826 (14)	0.0435 (3)	
H5	0.848114	1.031911	0.931918	0.052*	
C6	0.67633 (16)	0.89655 (16)	0.78049 (15)	0.0466 (3)	
C7	0.60415 (15)	0.84369 (14)	0.64163 (14)	0.0418 (3)	
H7	0.520189	0.775990	0.604414	0.050*	
C8	0.66952 (14)	0.61504 (12)	0.37752 (13)	0.0320 (2)	
H8A	0.569909	0.559766	0.327692	0.038*	
H8B	0.694307	0.631215	0.473573	0.038*	
C9	0.79009 (13)	0.53710 (12)	0.34387 (11)	0.0300 (2)	
C10	0.93273 (14)	0.60756 (12)	0.37335 (12)	0.0314 (2)	
C11	1.04486 (14)	0.53163 (14)	0.34808 (13)	0.0358 (3)	
C12	1.01851 (15)	0.38994 (14)	0.29476 (13)	0.0405 (3)	
H12	1.094355	0.340736	0.277973	0.049*	
C13	0.87734 (16)	0.32319 (13)	0.26702 (13)	0.0394 (3)	
C14	0.76347 (14)	0.39528 (12)	0.29014 (12)	0.0348 (3)	
H14	0.668492	0.348467	0.269575	0.042*	
C15	0.60508 (17)	0.73494 (15)	0.19605 (13)	0.0430 (3)	
H15A	0.659421	0.666455	0.159482	0.052*	0.867 (3)
H15B	0.633807	0.822342	0.184310	0.052*	0.867 (3)
H15C	0.690386	0.713498	0.170147	0.052*	0.133 (3)
H15D	0.579251	0.824618	0.181553	0.052*	0.133 (3)
Cl2A	0.5985 (10)	0.8517 (9)	0.8845 (5)	0.0813 (12)	0.49 (3)
O3	0.38914 (17)	0.56244 (17)	0.11430 (15)	0.0620 (5)	0.867 (3)
C16	0.4335 (2)	0.6951 (2)	0.11144 (19)	0.0510 (5)	0.867 (3)
H16A	0.376368	0.761397	0.146831	0.061*	0.867 (3)
H16B	0.409768	0.696484	0.018604	0.061*	0.867 (3)
C17	0.2289 (4)	0.5234 (5)	0.0411 (4)	0.0727 (10)	0.867 (3)
H17A	0.203010	0.508710	−0.054032	0.109*	0.867 (3)
H17B	0.174109	0.595322	0.070034	0.109*	0.867 (3)
H17C	0.200589	0.439687	0.057594	0.109*	0.867 (3)
Cl2B	0.6178 (8)	0.8240 (12)	0.8838 (5)	0.0852 (13)	0.51 (3)
O3A	0.3455 (10)	0.6544 (11)	0.1361 (8)	0.058 (2)	0.133 (3)
C16A	0.4692 (13)	0.6306 (17)	0.1002 (12)	0.055 (3)	0.133 (3)
H16C	0.441195	0.637306	0.008277	0.066*	0.133 (3)
H16D	0.496146	0.538240	0.103566	0.066*	0.133 (3)
C17A	0.215 (3)	0.552 (3)	0.065 (3)	0.090 (7)	0.133 (3)
H17D	0.159714	0.561535	−0.024522	0.135*	0.133 (3)
H17E	0.148807	0.563079	0.112909	0.135*	0.133 (3)
H17F	0.249224	0.461729	0.060152	0.135*	0.133 (3)

### Atomic displacement parameters (Å^2^)

**Table:**

	*U*^11^	*U*^22^	*U*^33^	*U*^12^	*U*^13^	*U*^23^
Cl1	0.0519 (2)	0.03981 (18)	0.0515 (2)	−0.01450 (15)	0.01056 (17)	0.00156 (15)
Cl3	0.03336 (16)	0.0688 (3)	0.0631 (2)	0.00036 (16)	0.02120 (16)	0.02153 (19)
Cl4	0.0699 (3)	0.03300 (18)	0.0738 (3)	0.00873 (17)	0.0259 (2)	−0.00547 (17)
O1	0.0605 (7)	0.0419 (5)	0.0461 (6)	−0.0170 (5)	0.0224 (5)	0.0031 (5)
O2	0.0371 (5)	0.0301 (4)	0.0494 (5)	−0.0045 (4)	0.0169 (4)	0.0057 (4)
N1	0.0347 (5)	0.0278 (5)	0.0325 (5)	0.0036 (4)	0.0138 (4)	0.0067 (4)
C1	0.0330 (6)	0.0336 (6)	0.0383 (6)	0.0054 (5)	0.0122 (5)	0.0042 (5)
C2	0.0310 (6)	0.0287 (5)	0.0387 (6)	0.0051 (5)	0.0139 (5)	0.0023 (5)
C3	0.0373 (6)	0.0274 (5)	0.0401 (6)	0.0030 (5)	0.0170 (5)	0.0044 (5)
C4	0.0344 (6)	0.0285 (6)	0.0425 (7)	0.0001 (5)	0.0125 (5)	0.0017 (5)
C5	0.0391 (7)	0.0475 (8)	0.0369 (7)	0.0012 (6)	0.0129 (6)	0.0034 (6)
C6	0.0402 (7)	0.0570 (9)	0.0432 (7)	−0.0030 (6)	0.0194 (6)	0.0103 (6)
C7	0.0334 (6)	0.0435 (7)	0.0441 (7)	−0.0033 (5)	0.0156 (6)	0.0044 (6)
C8	0.0326 (5)	0.0292 (5)	0.0344 (6)	0.0015 (4)	0.0145 (5)	0.0071 (5)
C9	0.0309 (5)	0.0290 (5)	0.0282 (5)	0.0015 (4)	0.0110 (4)	0.0060 (4)
C10	0.0314 (5)	0.0315 (6)	0.0280 (5)	−0.0004 (4)	0.0094 (4)	0.0068 (4)
C11	0.0293 (5)	0.0450 (7)	0.0330 (6)	0.0029 (5)	0.0121 (5)	0.0111 (5)
C12	0.0378 (6)	0.0477 (7)	0.0341 (6)	0.0128 (6)	0.0143 (5)	0.0079 (5)
C13	0.0448 (7)	0.0308 (6)	0.0354 (6)	0.0065 (5)	0.0119 (5)	0.0035 (5)
C14	0.0346 (6)	0.0305 (6)	0.0337 (6)	−0.0008 (5)	0.0110 (5)	0.0039 (5)
C15	0.0507 (8)	0.0445 (7)	0.0360 (7)	0.0067 (6)	0.0182 (6)	0.0135 (6)
Cl2A	0.080 (2)	0.106 (2)	0.0540 (16)	−0.0391 (15)	0.0384 (17)	−0.0025 (18)
O3	0.0505 (8)	0.0610 (10)	0.0552 (8)	−0.0033 (7)	0.0030 (6)	0.0130 (7)
C16	0.0561 (12)	0.0518 (11)	0.0344 (8)	0.0134 (9)	0.0077 (8)	0.0103 (8)
C17	0.0510 (13)	0.098 (3)	0.0480 (18)	−0.0115 (16)	0.0108 (11)	−0.0010 (15)
Cl2B	0.0609 (12)	0.139 (3)	0.0544 (16)	−0.0285 (17)	0.0132 (13)	0.042 (2)
O3A	0.053 (4)	0.069 (5)	0.038 (3)	−0.004 (4)	0.014 (3)	−0.001 (3)
C16A	0.054 (5)	0.068 (5)	0.038 (4)	0.010 (5)	0.016 (4)	0.007 (4)
C17A	0.065 (9)	0.102 (10)	0.051 (10)	0.007 (9)	−0.011 (8)	−0.013 (8)

### Geometric parameters (Å, º)

**Table:**

Cl1—C4	1.7284 (13)	C9—C14	1.3841 (16)
Cl3—C11	1.7324 (13)	C10—C11	1.3927 (17)
Cl4—C13	1.7337 (13)	C11—C12	1.3825 (19)
O1—H1	0.82 (2)	C12—H12	0.9300
O1—C3	1.3465 (16)	C12—C13	1.378 (2)
O2—H2	0.87 (2)	C13—C14	1.3789 (18)
O2—C10	1.3540 (14)	C14—H14	0.9300
N1—C1	1.4720 (15)	C15—H15A	0.9700
N1—C8	1.4752 (15)	C15—H15B	0.9700
N1—C15	1.4763 (16)	C15—H15C	0.9700
C1—H1A	0.9700	C15—H15D	0.9700
C1—H1B	0.9700	C15—C16	1.518 (2)
C1—C2	1.5085 (17)	C15—C16A	1.502 (12)
C2—C3	1.4002 (17)	O3—C16	1.405 (3)
C2—C7	1.3854 (19)	O3—C17	1.413 (4)
C3—C4	1.3906 (18)	C16—H16A	0.9700
C4—C5	1.381 (2)	C16—H16B	0.9700
C5—H5	0.9300	C17—H17A	0.9600
C5—C6	1.372 (2)	C17—H17B	0.9600
C6—C7	1.380 (2)	C17—H17C	0.9600
C6—Cl2A	1.732 (7)	O3A—C16A	1.386 (13)
C6—Cl2B	1.748 (6)	O3A—C17A	1.427 (16)
C7—H7	0.9300	C16A—H16C	0.9700
C8—H8A	0.9700	C16A—H16D	0.9700
C8—H8B	0.9700	C17A—H17D	0.9600
C8—C9	1.5079 (16)	C17A—H17E	0.9600
C9—C10	1.4015 (16)	C17A—H17F	0.9600
			
C3—O1—H1	111.2 (14)	C13—C12—C11	118.54 (12)
C10—O2—H2	105.1 (13)	C13—C12—H12	120.7
C1—N1—C8	111.85 (10)	C12—C13—Cl4	119.75 (10)
C1—N1—C15	112.00 (10)	C12—C13—C14	121.22 (12)
C8—N1—C15	113.80 (10)	C14—C13—Cl4	119.03 (11)
N1—C1—H1A	109.0	C9—C14—H14	119.9
N1—C1—H1B	109.0	C13—C14—C9	120.21 (12)
N1—C1—C2	113.07 (10)	C13—C14—H14	119.9
H1A—C1—H1B	107.8	N1—C15—H15A	108.0
C2—C1—H1A	109.0	N1—C15—H15B	108.0
C2—C1—H1B	109.0	N1—C15—H15C	107.6
C3—C2—C1	119.30 (12)	N1—C15—H15D	107.6
C7—C2—C1	121.13 (11)	N1—C15—C16	117.22 (13)
C7—C2—C3	119.50 (12)	N1—C15—C16A	118.7 (6)
O1—C3—C2	116.78 (12)	H15A—C15—H15B	107.2
O1—C3—C4	124.53 (12)	H15C—C15—H15D	107.1
C4—C3—C2	118.69 (12)	C16—C15—H15A	108.0
C3—C4—Cl1	120.12 (10)	C16—C15—H15B	108.0
C5—C4—Cl1	118.21 (10)	C16A—C15—H15C	107.6
C5—C4—C3	121.67 (12)	C16A—C15—H15D	107.6
C4—C5—H5	120.6	C16—O3—C17	112.0 (3)
C6—C5—C4	118.72 (13)	C15—C16—H16A	109.6
C6—C5—H5	120.6	C15—C16—H16B	109.6
C5—C6—C7	121.17 (13)	O3—C16—C15	110.13 (15)
C5—C6—Cl2A	118.2 (2)	O3—C16—H16A	109.6
C5—C6—Cl2B	119.8 (2)	O3—C16—H16B	109.6
C7—C6—Cl2A	120.2 (2)	H16A—C16—H16B	108.1
C7—C6—Cl2B	118.9 (3)	O3—C17—H17A	109.5
C2—C7—H7	119.9	O3—C17—H17B	109.5
C6—C7—C2	120.26 (13)	O3—C17—H17C	109.5
C6—C7—H7	119.9	H17A—C17—H17B	109.5
N1—C8—H8A	109.4	H17A—C17—H17C	109.5
N1—C8—H8B	109.4	H17B—C17—H17C	109.5
N1—C8—C9	111.23 (10)	C16A—O3A—C17A	115.8 (15)
H8A—C8—H8B	108.0	C15—C16A—H16C	109.7
C9—C8—H8A	109.4	C15—C16A—H16D	109.7
C9—C8—H8B	109.4	O3A—C16A—C15	109.7 (10)
C10—C9—C8	119.85 (10)	O3A—C16A—H16C	109.7
C14—C9—C8	120.31 (10)	O3A—C16A—H16D	109.7
C14—C9—C10	119.77 (11)	H16C—C16A—H16D	108.2
O2—C10—C9	120.78 (11)	O3A—C17A—H17D	109.5
O2—C10—C11	120.70 (11)	O3A—C17A—H17E	109.5
C11—C10—C9	118.52 (11)	O3A—C17A—H17F	109.5
C10—C11—Cl3	119.57 (10)	H17D—C17A—H17E	109.5
C12—C11—Cl3	118.68 (10)	H17D—C17A—H17F	109.5
C12—C11—C10	121.74 (11)	H17E—C17A—H17F	109.5
C11—C12—H12	120.7		

### Hydrogen-bond geometry (Å, º)

**Table:**

*D*—H···*A*	*D*—H	H···*A*	*D*···*A*	*D*—H···*A*
O1—H1···Cl1	0.82 (2)	2.58 (2)	3.0455 (12)	117.4 (17)
O2—H2···N1	0.87 (2)	1.83 (2)	2.6365 (16)	153 (2)
O1—H1···O2^i^	0.82 (2)	2.01 (2)	2.7709 (14)	152.5 (19)

Symmetry code: (i) −*x*+2, −*y*+2, −*z*+1.
